# Glucocerebrosidase 1 deficient *Danio rerio* mirror key pathological aspects of human Gaucher disease and provide evidence of early microglial activation preceding alpha-synuclein-independent neuronal cell death

**DOI:** 10.1093/hmg/ddv369

**Published:** 2015-09-16

**Authors:** Marcus Keatinge, Hai Bui, Aswin Menke, Yu-Chia Chen, Anna M. Sokol, Qing Bai, Felix Ellett, Marc Da Costa, Derek Burke, Matthew Gegg, Lisa Trollope, Thomas Payne, Aimee McTighe, Heather Mortiboys, Sarah de Jager, Hugh Nuthall, Ming-Shang Kuo, Angeleen Fleming, Anthony H.V. Schapira, Stephen A. Renshaw, J. Robin Highley, Agnieszka Chacinska, Pertti Panula, Edward A. Burton, Michael J. O'Neill, Oliver Bandmann

**Affiliations:** 1 The Bateson Centre,; 2 Sheffield Institute for Translational Neuroscience (SITraN),; 3 Department of Infection and Immunity, University of Sheffield, Sheffield, UK,; 4 Lilly Research Laboratories, Eli Lilly & Company, Indianapolis, USA,; 5 TNO, Zeist, The Netherlands,; 6 Neuroscience Center and Department of Anatomy, University of Helsinki, Finland,; 7 Laboratory of Mitochondrial Biogenesis, International Institute of Molecular and Cell Biology, Warsaw, Poland,; 8 Pittsburgh Institute for Neurodegenerative Diseases and Department of Neurology, University of Pittsburgh School of Medicine, Pittsburgh, USA,; 9 Molecular and Genetics Unit, University College London Institute of Child Health,; 10 Enzyme Unit and Metabolic Unit, Chemical Pathology, Great Ormond Street Hospital, London, UK,; 11 Department of Clinical Neurosciences, University College London Institute of Neurology, London, UK,; 12 Department of Medical Genetics, Cambridge Institute for Medical Research University of Cambridge, Cambridge, UK and; 13 Eli Lilly and Company Limited, Surrey, UK

## Abstract

Autosomal recessively inherited *glucocerebrosidase 1* (*GBA1*) mutations cause the lysosomal storage disorder Gaucher's disease (GD). Heterozygous *GBA1* mutations (*GBA1*^+/−^) are the most common risk factor for Parkinson's disease (PD). Previous studies typically focused on the interaction between the reduction of glucocerebrosidase (enzymatic) activity in *GBA1*^+/−^ carriers and alpha-synuclein-mediated neurotoxicity. However, it is unclear whether other mechanisms also contribute to the increased risk of PD in *GBA1^+/−^* carriers. The zebrafish genome does not contain *alpha-synuclein* (*SNCA*), thus providing a unique opportunity to study pathogenic mechanisms unrelated to alpha-synuclein toxicity. Here we describe a mutant zebrafish line created by TALEN genome editing carrying a 23 bp deletion in *gba1* (*gba1^c.1276_1298del^*), the zebrafish orthologue of human *GBA1*. Marked sphingolipid accumulation was already detected at 5 days post-fertilization with accompanying microglial activation and early, sustained up-regulation of miR-155, a master regulator of inflammation. *gba1^c.1276_1298del^* mutant zebrafish developed a rapidly worsening phenotype from 8 weeks onwards with striking reduction in motor activity by 12 weeks. Histopathologically, we observed marked Gaucher cell invasion of the brain and other organs. Dopaminergic neuronal cell count was normal through development but reduced by >30% at 12 weeks in the presence of ubiquitin-positive, intra-neuronal inclusions. This *gba1^c.1276_1298del^* zebrafish line is the first viable vertebrate model sharing key pathological features of GD in both neuronal and non-neuronal tissue. Our study also provides evidence for early microglial activation prior to alpha-synuclein-independent neuronal cell death in GBA1 deficiency and suggests upregulation of miR-155 as a common denominator across different neurodegenerative disorders.

## Introduction

Gaucher's disease (GD) is the most common lysosomal storage disorder with a prevalence of 1:40 000 (1). It is caused by autosomal recessively inherited homozygous or compound heterozygous mutations in *glucocerebrosidase 1* (*GBA1*). GBA1 is a lysosomal enzyme required for the breakdown of glucosylceramide to ceramide and glucose and forms part of the sphingolipid pathway. The pathological hallmark of GD is the accumulation of characteristic macrophages engorged with glycolipids also known as Gaucher cells. Clinically, GD can present heterogeneously with three different subtypes, categorized by severity and distribution of symptoms. Patients with type I can be virtually asymptomatic, type II presents with rapid neurological decline and subsequent death within the first 3 years of life, whereas type III presents with neurological decline during adolescence (2). Current treatment options largely focus on enzyme replacement therapy, which is effective for the treatment of non-neurological complications of GD but ineffective for the treatment or prevention of neurological complications due to its inability to cross the blood–brain barrier (3).

Heterozygous *GBA1* mutations (*GBA1^+/−^*) are the most common risk factor for Parkinson's disease (PD) with an odds ratio of >5 (4–6). PD patients carrying such a heterozygous *GBA1* mutation have an earlier age of onset and are more likely to develop impaired cognitive function (7,8).

Both toxic gain of function and loss of function mechanisms have been proposed to explain the link between heterozygous *GBA1* mutations and PD with particular focus on an interaction between glucocerebrosidase 1 (GCase) enzymatic activity and alpha-synuclein (6,9).

*GBA1* knock out (KO) mouse die shortly after birth due to skin defects leading to a loss of hydration. Conditional *GBA1* KO mice with isolated neuronal GCase deficiency have an initial, symptom-free period of 10 days, followed by rapid neurological decline and subsequent death due to excessive seizures. Conditional KO mice in the hematopoietic and mesenchymal cell lineages model the major visceral symptoms of GD, but otherwise have a normal life span and fail to model the neuropathic forms (10).

Zebrafish have become a versatile disease model for studying neurodegeneration (11). As vertebrates, they are more closely related to humans than *Drosophila* or *Caenorhabditis elegans*, develop externally and are transparent. We and others have previously demonstrated their usefulness to identify novel drug targets in zebrafish models of PD and other neurodegenerative disorders (12,13).

We have used the TALEN (transcription activator-like effector nucleases) approach to create a *gba1* mutant zebrafish. Homozygous *gba1* mutant zebrafish (*gba1*^−/−^) develop normally but already display sphingolipid dysregulation and accumulation as early as 5 days post-fertilization (dpf) with marked alterations of the GD biomarkers β-hexosaminidase and chitotriosidase in juvenile brain tissue. We further demonstrate early microglial activation with marked upregulation of miRNA-155 (miR-155) which precedes subsequent organ infiltration with Gaucher cells in juvenile *gba1^−/−^*. These *gba1^−/−^* zebrafish also develop progressive neurodegeneration, mitochondrial dysfunction and loss of dopaminergic neurons with ubiquitin-positive inclusions in the absence of alpha-synuclein. This new vertebrate model of GCase deficiency is likely to have utility for future gene–gene interaction studies and *in vivo* drug screens. The identification of distinct and potentially ‘druggable’ molecular targets such as miR-155 will facilitate these *in vivo* drug screens.

## Results

### Zebrafish possess a single GBA1 orthologue

**Figure 1.**
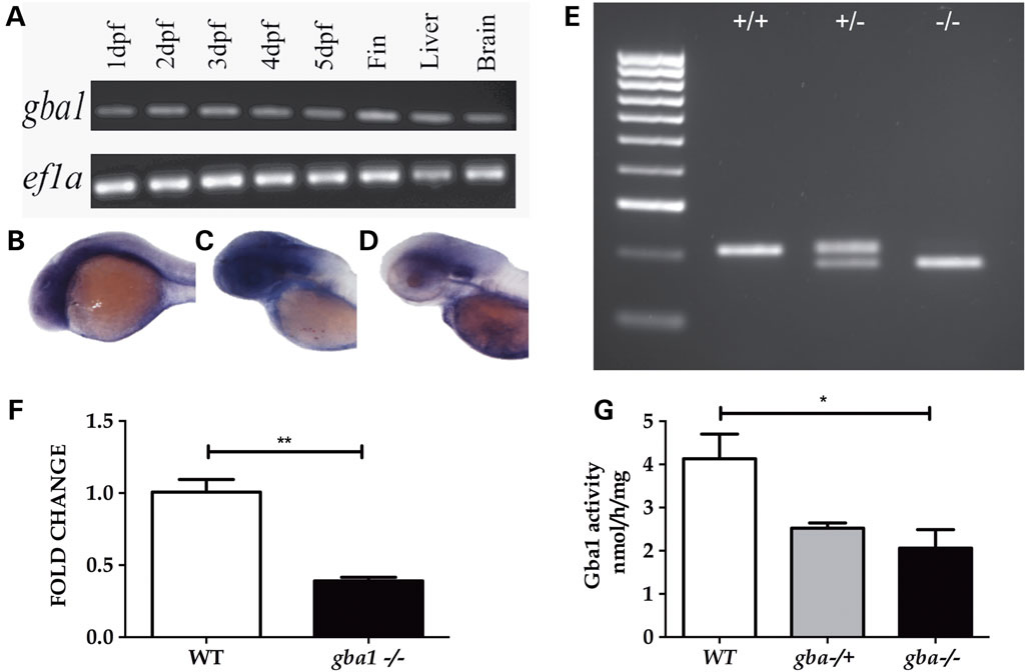
*gba1* expression in wild-type (WT) zebrafish and loss of function studies. *gba1* expression through early development and in adult organs particularly affected by GD (namely brain and liver) was confirmed by RT–PCR (**A**); *ef1a* was used as a loading control. WISH confirmed early expression of *gba1* in brain tissue at 1 dpf (**B**), 2 dpf (**C**) and 3 dpf (**D**). Using TALENs, a 23 bp deletion in exon 7 of *gba1* (*gba1^c.1276_129del^*) was generated which could be genotyped by PCR. A representative genotyping gel (**E**) shows WT (lane 1), *gba1^+/−^* (lane 2) and *gba1^−/−^* (lane 3). The *gba1^c.1276_129del^* mutation resulted in a >50% decrease in *gba1* transcript levels in *gba1^−/−^* brain tissue (*P* < 0.01, **F**) and a decrease in enzymatic GCase activity (*P* < 0.05, **G**). *P < 0.05; **P < 0.01.

A BLAST search identified a single zebrafish orthologue of human *GBA1* on chromosome 16 (ENSDARG00000076058) of the zebrafish genome. The zebrafish gene (*gba1*) encodes a single protein of 518 amino acids and 57% identity with the human orthologue. The genetic loci of both (human) *GBA1* and (*Danio rerio*) *gba1* shared conserved synteny, both containing the genes *RUSC1*, *FDPS* and *DAP3* within 500 kb of each orthologue. *gba1* was expressed at constant levels through 1–5 dpf with more marked expression in the brain. Expression was also detected in adult brain and liver tissue, organs specifically affected by GD pathology (Fig. 1A–D).

### *gba1* TALEN-generated mutants are loss of function

Using TALEN technology, we generated a *gba1* mutant containing a 23 bp deletion in exon 7 (c.1276_1298del, Fig. 1E and Supplementary Material, Fig. S1). The deletion results in a frame-shift at position c.1276 and a subsequent premature stop codon 66 bp downstream, within exon 7 at c.1342 (p.379). The *gba1^c.1276_1298del^* (from hereon referred to as *gba1^−/−^*) resulted in a reduction of *gba1* mRNA by >50% (*P* < 0.01, Fig. 1F). Similarly, GCase activity was reduced in *gba1*^−/−^ brains by >50% (*P* < 0.05) compared with wild-type (Fig. 1G).

### Analysis of sphingolipid metabolites

**Figure 2.**
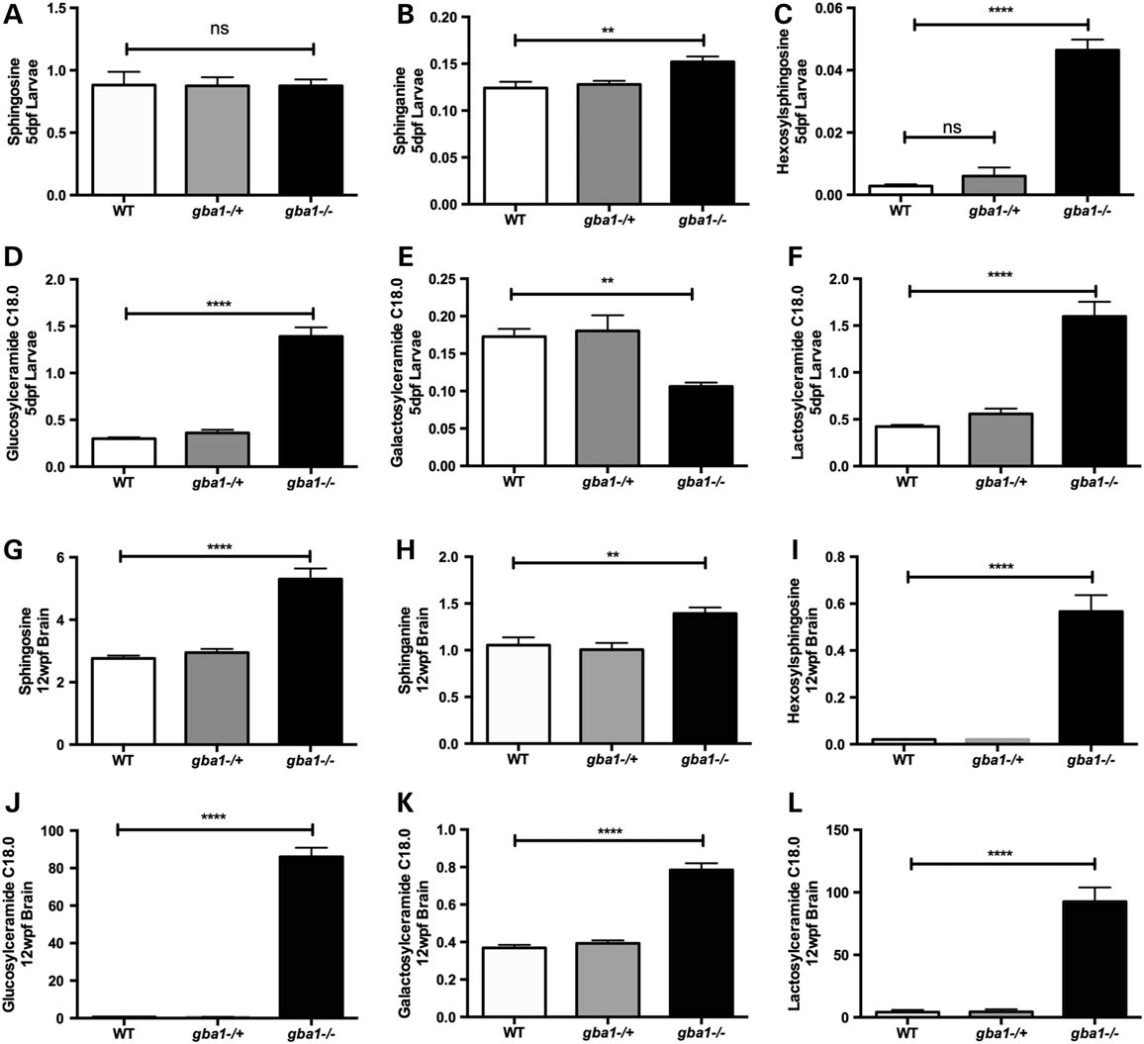
Sphingolipid metabolites accumulate in zebrafish larvae and brain tissue. Sphingolipid metabolites were analyzed across *gba1* genotypes in 5 dpf larvae and 12 wpf brain tissue. Sphingosine levels remained unaltered across all genotypes at 5 dpf (**A**) but were approximately doubled in *gba1^−/−^* brains at 12 wpf (**G**). In contrast, sphinganine (**B**), hexosylsphingosine (**C**), glucosylceramide C18.0 (**D**) and lactosylceramide C18.0 (**F**) had already accumulated in *gba1^−/−^* larvae up to 1500% of control values, whereas galactosylceramide C18.0 (**E**) levels were reduced by 50%. In 12 wpf brain tissue, all these sphingolipid metabolites had accumulated in *gba1^−/−^* by up to 14 000% (**G–L**). The concentration of each sphingolipid is given in ng/mg (ns: non-significant; **P < 0.01; ****P < 0.0001).

GCase deficiency leads to marked sphingolipid dysregulation and accumulation of GCase substrates in *Gba1* KO mice and patients with GD (14–16). We analyzed sphingolipid metabolites by mass spectrometry across all *gba1* genotypes and identified marked accumulation of sphingolipid metabolites as early as 5 dpf in *gba1^−/−^*, with increases in the C18 molecular weight species of each glycolipid being the most pronounced (Fig. 2). Hexosylsphingosine was virtually undetectable in wild-type samples but increased to 1573% in *gba1^−/−^* of the level seen in controls (Fig. 2C; *P* < 0.0001), glucosylceramide was increased to ∼360% (Fig. 2D; *P* < 0.0001). Substrates upstream of GCase also accumulated, namely lactosylceramide to nearly 300% (Fig. 2F; *P* < 0.0001) whereas galactosylceramide was notably decreased by 50% (Fig. 2E; *P* < 0.01). Mass spectrometric analysis was repeated in juvenile brain tissue at 12 weeks post-fertilization (wpf) across all *gba1* genotypes. Again, direct substrates of GCase had the largest increases in *gba1^−/−^* brains: hexosylsphingosine was virtually undetectable in wild-type brains but increased in *gba1^−/−^* to 2734% of the level seen in controls (Fig. 2I; *P* < 0.0001), whereas glucosylceramide increased to 14 000% (Fig. 2J; *P* < 0.0001). Galactosylceramide was now increased as well (Fig. 2K; *P* < 0.0001) but not as strongly as lactosylceramides which increased above wild-type levels to 2000% of the level seen in controls (Fig. 2L; *P* < 0.0001). Sphingosine levels were unaltered in 5 dpf larval homogenates but doubled in *gba1^−/−^* juvenile brains (Fig. 2A and G, *P* < 0.0001), sphinganine levels were increased to a similar extent in *gba1^−/−^* larvae and juvenile brains (Fig. 2B and H, *P* < 0.01). In contrast, there were no significant changes for any of the analyzed sphingolipid metabolites in either *gba1^+/−^* larvae or *gba1^+/−^* juvenile brains compared with wild-type (see also Supplementary Material, Table S1 which lists all metabolites analyzed).

### *gba1*^−/−^ zebrafish mirror key Gaucher's disease phenotypes

**Figure 3.**
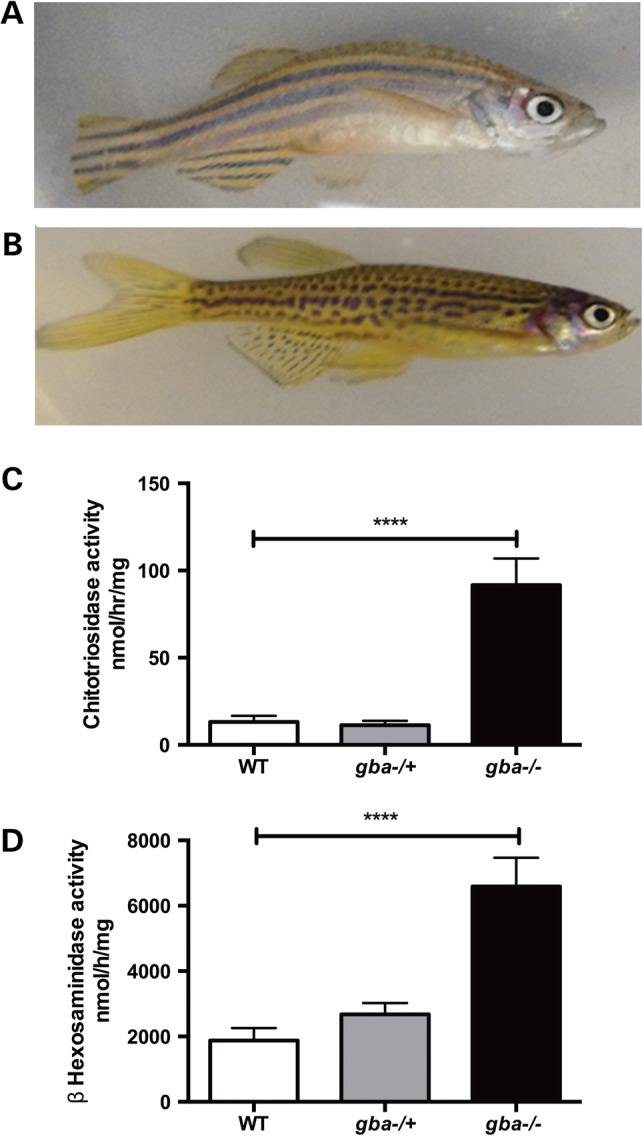
Skeletal and biochemical indices of GCase deficiency. At 12 wpf *gba1^−/−^* (**A**) developed a curve to their spine compared with wild-type (WT) controls (**B**) in a similar manner to some conditional *GBA1* KO mice (17). The difference in the stripe pattern of *gba1^−/−^* (A) and wild-type zebrafish (B) is due to the *gba1^+/−^* line being of mixed lineage with (wild-type) AB and (wild-type) TL genetic background. Classical Gaucher disease biomarkers were markedly elevated in *gba1^−/−^* brains at 12 wpf, with a 10-fold increase in chitotriosidase activity (*P* < 0.0001) (**C**) and a 4-fold increase in β-hexosaminidase activity (*P* < 0.0001) (**D**). No significant changes were detected in either assay in *gba1^+/−^* brains. ****P <0.0001.

*gba1*^−/−^ and *gba1*^+/−^ did not develop an overt morphological phenotype during early development. By 8 wpf, *gba1*^−/−^ first began to swim more slowly and to generally look less well. By 12 wpf, juvenile *gba1*^−/−^ developed a curvature of the spine, reminiscent of the gibbus formation seen in conditional mouse KO models (Fig. 3A and B) (17). The oldest *gba1*^−/−^ fish reached an age of 14 wpf before death during pilot longevity studies. Consequently, all *gba1*^−/−^ fish were culled at 12 wpf for humane reasons.

Chitotriosidase and β-hexosaminidase activity are markedly increased in the serum of GD patients and used as biomarkers to monitor disease activity (1). In *gba1*^−/−^ zebrafish brains, chitotriosidase activity was increased ∼10-fold in *gba1^−/−^* brains (*P* < 0.0001; Fig. 3C) without a change in *gba1*^+/−^. Similarly, β-hexosaminidase activity was increased to 350% of values observed in controls (*P* < 0.0001) at 12 wpf but no difference was observed in *gba1*^+/−^ brains (*P* > 0.05; Fig. 3D). In contrast, β-galactosidase activity remained unchanged in its activity across all genotypes (data not shown).

**Figure 4.**
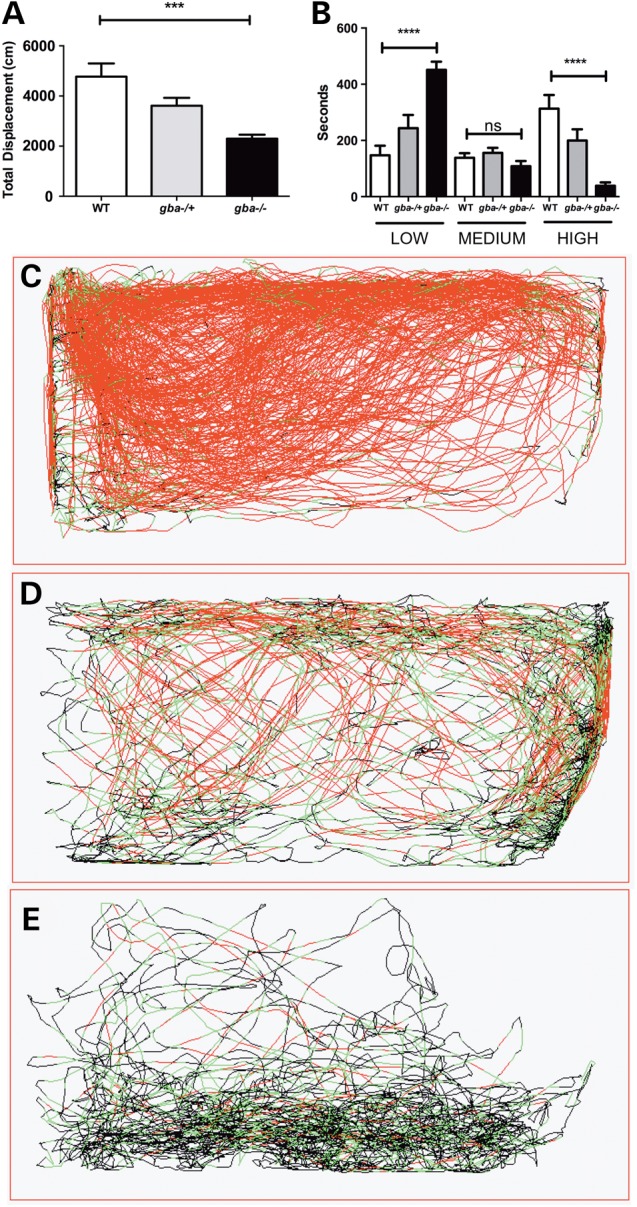
Marked slowing of spontaneous motor activity in *gba1^−/−^*. Video-tracking software was utilized to measure locomotion in *gba1* genotypes. All fish were filmed from the side. *gba1^−/−^* exhibited a 50% decrease in total displacement (**A**) (*P* < 0.001). When speeds were segregated into small medium and high speeds (**B**), *gba1^−/−^* spent more time moving at low speeds (300%, *P* < 0.0001) and a spent less time moving at high speeds (88% less, *P* < 0.0001). For representative movements traces of wild-type (WT) (**C**), *gba1^+/−^* (**D**) and *gba1^−/−^* (**E**), red lines represent high-speed movements, green represents medium-speed movements and black represents low-speed movements. ns, *P* >0.05; ***P < 0.001; ****P < 0.0001.

At 12 wpf, *gba1*^−/−^ showed a reduction in total displacement of 50% (*P* < 0.001), with a reduction by 25% in *gba1*^+/−^ (*P* > 0.05) (Fig. 4A). When individual swimming movements were assigned to low, medium and high speeds, wild-type fish spent the majority of their time making fast movements (Fig. 4B and C). The opposite was true of *gba1*^−/−^ fish, which spent most of their time making slow movements or remaining stationary (*P* < 0.0001, Fig. 4B and E). *gba1*^+/−^ fish had an intermediate phenotype for all speeds, but these changes were not significantly different to either wild-type or *gba1*^−/−^ (Fig. 4B and D). In addition, there were obvious defects of balance, with the *gba1^−/−^* animals showing severe variability of vertical body axis orientation (roll) during swimming, resulting in a ‘corkscrew’ pattern of motion. Occasional episodes were observed in which *gba1^−/−^* animals showed bursts of high-velocity movements, often violently moving in circles. These abnormalities were frequently interrupted by longer periods of inactivity during which the *gba1^−/−^* zebrafish lay on the tank floor (Supplementary Material, Video). These abnormalities were not seen in any of the heterozygous or wild-type sibling controls.

### *gba1^−/−^* exhibit Gaucher cell organ invasion and microglial activation

**Figure 5.**
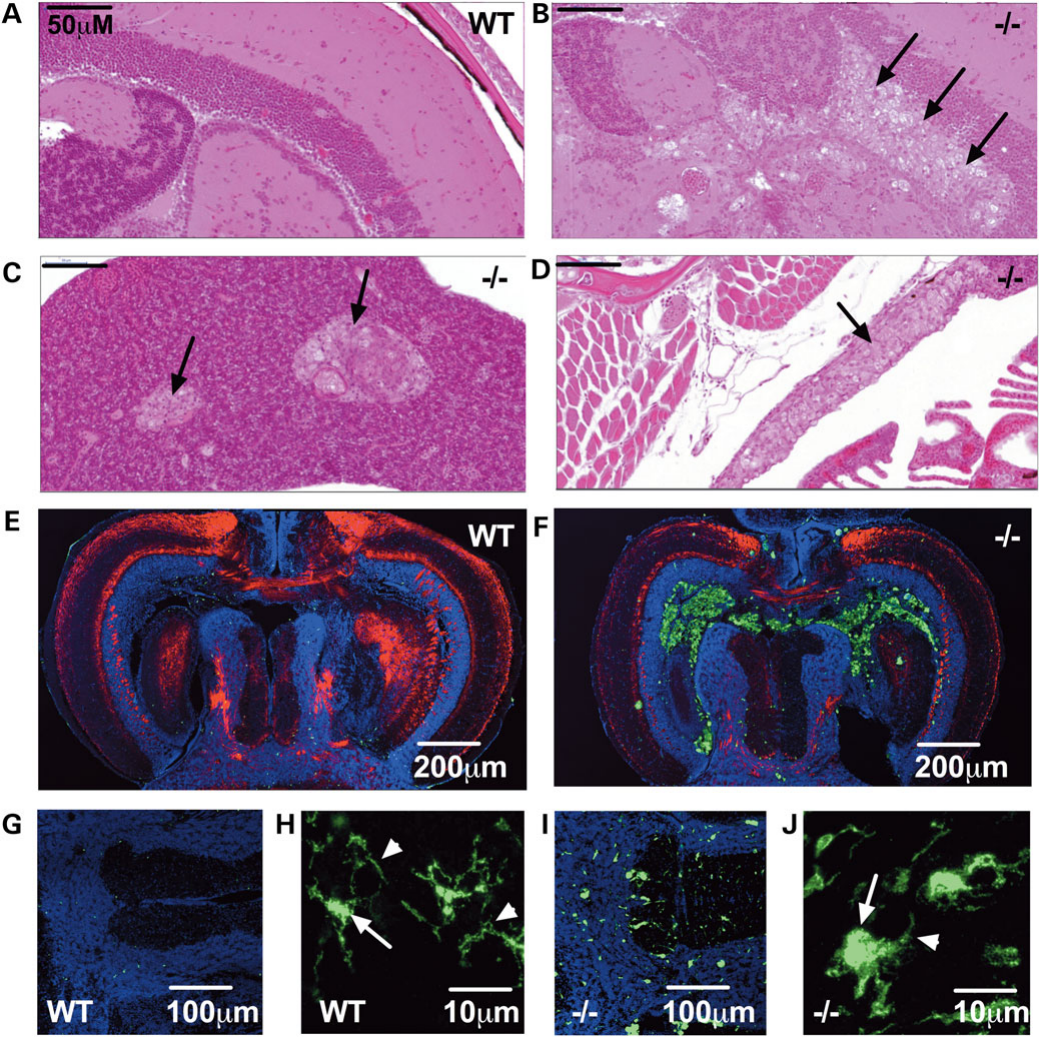
*gba1* deficiency leads to Gaucher cell invasion and increased abundance of activated microglia in *gba1^−/−^* brain. H&E staining of 12 wpf sections demonstrated Gaucher cell (black arrows) organ invasion of the tectal ventricle within the brain of *gba1^−/−^* (**B**), compared with wild-type (WT) brains (**A**). Gaucher cell organ invasion was also present in the visceral organs of *gba1^−/−^* such as the liver (**C**) and thymus (**D**). Fluorescent images show confocal micrographs of *gba1^−/−^* and wild-type siblings control brains labeled by indirect immunofluorescence for 4.C4 (macrophages and microglia; green), DAPI (nuclei; blue) and P0 (myelin; red). Low-power images through the tectal ventricle showed accumulation of 4.C4 immunoreactive macrophages in the ventricle and periventricular region of *gba1^−/−^* (**F**) but not wild-type brain (**E**). Microglia within the brain parenchyma were identified by their immunoreactivity to 4.C4 and typical morphology (**G** and **I**). Compared with wild-type brain (G), microglial were more numerous and brightly labeled in *gba1^−/−^* brain (I). In addition, compared with the typical quiescent morphology of microglia seen in wild-type brain (**H**), marked rounding of the cell body and retraction of processes was apparent in *gba1^−/−^* brain (**J**). The white arrows point at a normal microglial cell body in a wild-type control brain (H) and a rounded microglial cell body in a *gba1^−/−^* brain (J); the white arrow heads point at a normal, extended microglial process in a wild-type control brain (H) and at a retracted microglial process in a *gba1^−/−^* brain (J). These morphological changes are typical of microglial activation.

The primary histopathological hallmark of GD is the formation and accumulation of lipid-engorged macrophages known as Gaucher cells leading to visceral organomegaly. Microglial activation and other immune mechanisms have also been implicated in the pathogenesis of neuronal cell death in both GD and PD (18–20). Hematoxylin and eosin (H&E) staining in 12 wpf *gba1*^−/−^ revealed marked infiltration with enlarged ‘Gaucher-like’ cells not only in the brain (Fig. 5B), but also in liver (Fig. 5C), thymus (Fig. 5D) and pancreas (data not shown). As expected, no overt pathology could be detected in wild-type control individuals (Fig. 5A). Gaucher cells were periodic acid Schiff (PAS)-positive, indicative of glycolipid accumulation (data not shown). No abnormalities could be detected in the wild-type or *gba1*^+/−^ fish. There was no overt pathology at all in any of the three genotypes at 4 wpf (data not shown).

These Gaucher-like cells around the tectal ventricle labeled strongly with the 4.C4 monoclonal antibody marker for zebrafish monocyte/macrophage lineage cells (Fig. 5E and F). In addition, there was a marked increase in microglial cells in the brain parenchyma of *gba1^−/−^* zebrafish compared with controls (Fig. 5G and I). The microglia in *gba1^−/−^* brains showed swollen cell bodies and retracted processes typical of microglial activation (Fig. 5H and J).

**Figure 6.**
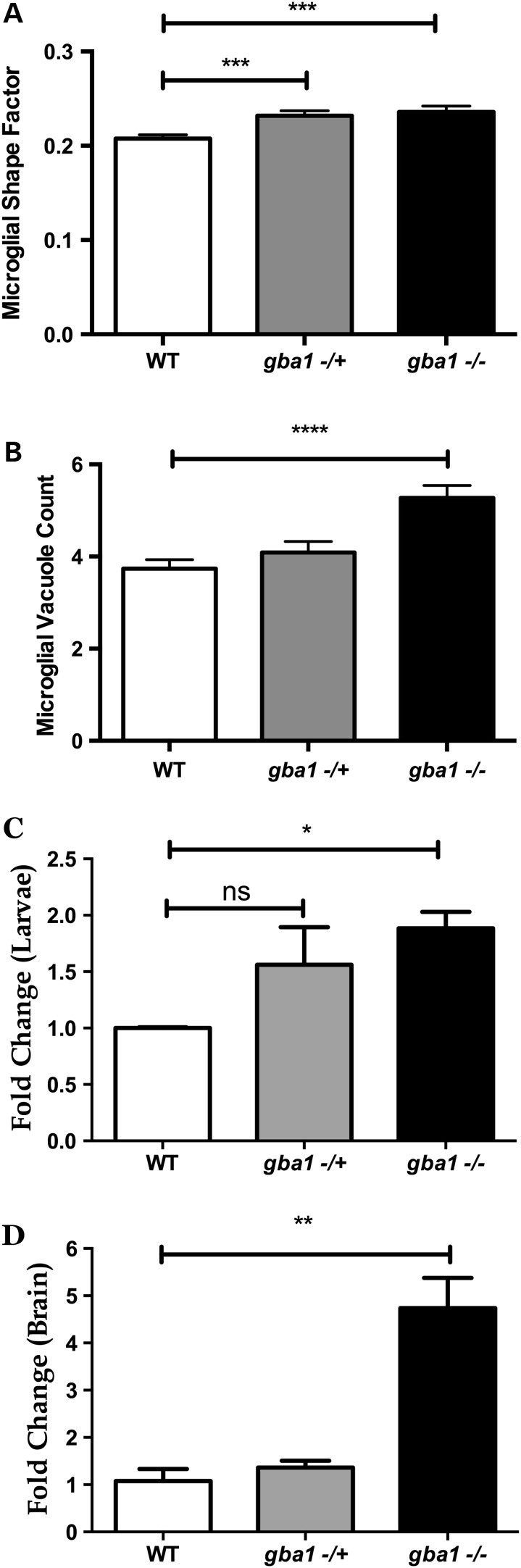
Activation of inflammatory/immune mechanisms during larval stages. Microglia in 4 dpf *gba1^+/−^* and *gba1^−/−^* had an increase in shape factor (sphericity) (*P* < 0.001 for both) indicative of microglial activation (**A**). Additionally, the number of vacuoles in *gba1^−/−^* was increased by 40% compared with the values observed in wild-type (WT) controls (*P* < 0.0001) (**B**). Levels of miR-155, a master regulator of inflammatory/immune mechanisms, were analyzed in 5 dpf larvae (**C**) and 12 wpf brain tissue (**D**) across *gba1* genotypes. miR-155 was increased 2-fold in *gba1^−/−^* larvae (*P* < 0.05) and 4-fold in 12 wpf *gba1^−/−^* brains (*P* < 0.01). *P < 0.05; **P < 0.01; ***P < 0.001; ****P < 0.0001.

The transparent nature of zebrafish embryos allows the assessment of microglial activation *in vivo* in a zebrafish transgenic line in which the membrane-targeted fluorescent reporter (GFP-CAAX) expression is driven by the promoter of macrophage-expressed gene 1 (*mpeg1*). We crossed this *mpeg1:*GFP-CAAX transgenic line with *gba1*^+/−^ zebrafish and then assessed microglial activation in larvae at 4 dpf across the three different genotypes to further determine whether altered immune mechanisms may precede overt neuropathology. *gba1^+/−^* and *gba1^−/−^* had altered microglial shape, reflecting microglial activation (shape factor in wild-type controls: 0.2077; *gba1^+/−^*: 0.2319; *gba1^−/−^*: 0.2356; *P* < 0.001 for both *gba1^+/−^* and *gba1^−/−^*; Fig. 6A). Microglia vacuole count was also increased in *gba1^−/−^* microglia by 40% with average count across genotypes being 3.737 (wild-type), 4.015 (*gba1^+/−^*, *P* > 0.05) and 5.273 (*gba1^−/−^*, *P* < 0.0001) per microglia (Fig. 6B). In contrast, microglia volume and absolute count were unchanged across the three genotypes (data not shown). miR-155 is a key regulator of inflammation (21). We hypothesized that miR-155 up-regulation may be an early feature in *gba1^−^*^/−^. As predicted, miR-155 levels were increase by 88% in *gba1^−/−^* larvae at 5 dpf compared with values observed in controls (*P* < 0.05, Fig. 6C), with an even more marked increase by 470% in juvenile *gba1*^−/−^ brain tissue (*P* < 0.01, Fig. 6D).

### *gba1^−/−^* undergo alpha-synuclein-independent neurodegeneration

**Figure 7.**
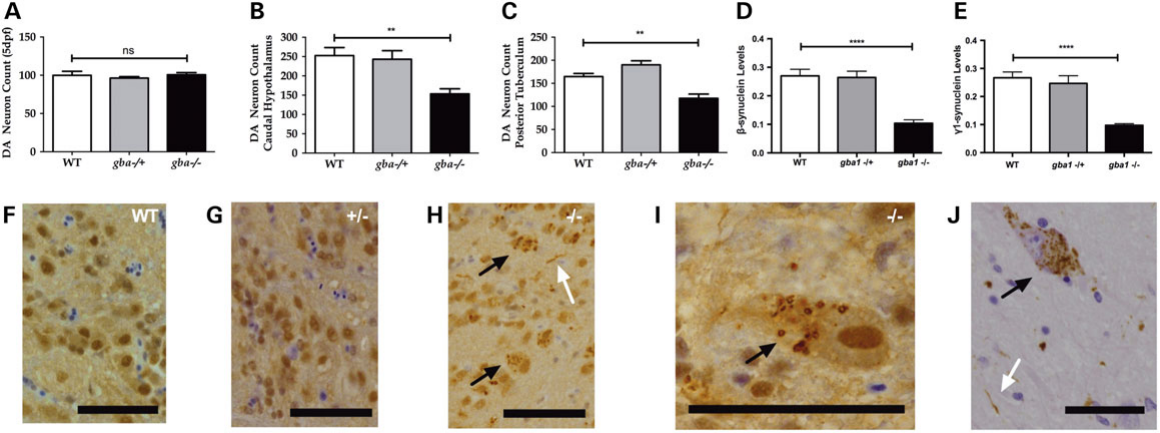
Dopaminergic neuronal cell loss and ubiquitinated inclusions in *gba1^−/−^* brains. The number of ascending diencephalic dopaminergic neurons (Rink–Wullimann groups 1, 2, 4 and 5) was similar across the three *gba1* genotypes at 5 dpf (**A**). In contrast, there was a 40% loss (*P* < 0.01) of the dopaminergic neurons in the caudal hypothalamus (**B**) and a 30% loss (*P* < 0.01) in the posterior tuberculum at 12 wpf (**C**). β (**D**) and γ1 (**E**) synuclein proteins levels were reduced by 60% in *gba1^−/−^* brain tissue suggesting a distinct loss of synapses due to global neurodegeneration. **F–J**: IHC labels ubiquitin brown by 3,3′-DAB. Glial cell nuclei are highlighted by hematoxylin counterstaining (blue). At 12 weeks of age, there is no significant pathology in wild-type (WT) (F) or *gba1^+/−^* fish (G). In contrast, *gba1^−/−^* fish (H and I) have granular ubiquitylated neuronal cytoplasmic inclusions (black arrows) and ubiquitylated neuritic pathology (white arrow). These granular neuronal cytoplasmic inclusions and neurites resemble the granular aggregates of a-synuclein (black arrow) and Lewy neurites (white arrow) as seen in sporadic PD (J; substantia nigra) (scale bar = 50 μm throughout). ns, *P* > 0.05; **P < 0.01; ****P < 0.0001.

The *alpha-synuclein* (*SNCA*) gene is notably absent in the zebrafish genome, but zebrafish possess orthologues of *beta*- and *gamma-synuclein* (22). To further investigate the effect of partial or complete GCase deficiency in the absence of alpha-synuclein (protein), dopaminergic neuronal cells were counted both during development and in juvenile zebrafish. At 5 dpf, there was no difference between either *gba1*^+/−^ or *gba1^−^*^/−^ and wild-type controls in the number of ascending dopaminergic neurons within the posterior tuberculum, the anatomical structure in zebrafish analogous to the human *substantia nigra pars compacta* (Fig. 7A). By 12 wpf, however, there was a marked reduction of dopaminergic neurons in both the caudal hypothalamus by 40% (*P* < 0.01; Fig. 7B) and the posterior tuberculum by ∼30% (*P* < 0.01; Fig. 7C). These data show unequivocally that dopaminergic neurons degenerate in *gba1^−/−^* zebrafish. Unexpectedly, both beta- and gamma-synuclein protein levels were markedly reduced by 60% in *gba1*^−/−^ brains (*P* < 0.0001; Fig. 7D and E). Microscopically, there was an abundance of ubiquitylated neuronal cytoplasmic inclusions as well as occasional ubiquitylated neurites throughout the CNS, but most prominently in the larger hindbrain neurons of *gba1*^−/−^ fish at 12 wpf (Fig. 7H and I) which bear resemblance to Lewy bodies and Lewy neurites in postmortem PD brain tissue (Fig. 7J).

### *gba1^−/−^*-induced mitochondrial dysfunction and impaired autophagy

**Figure 8.**
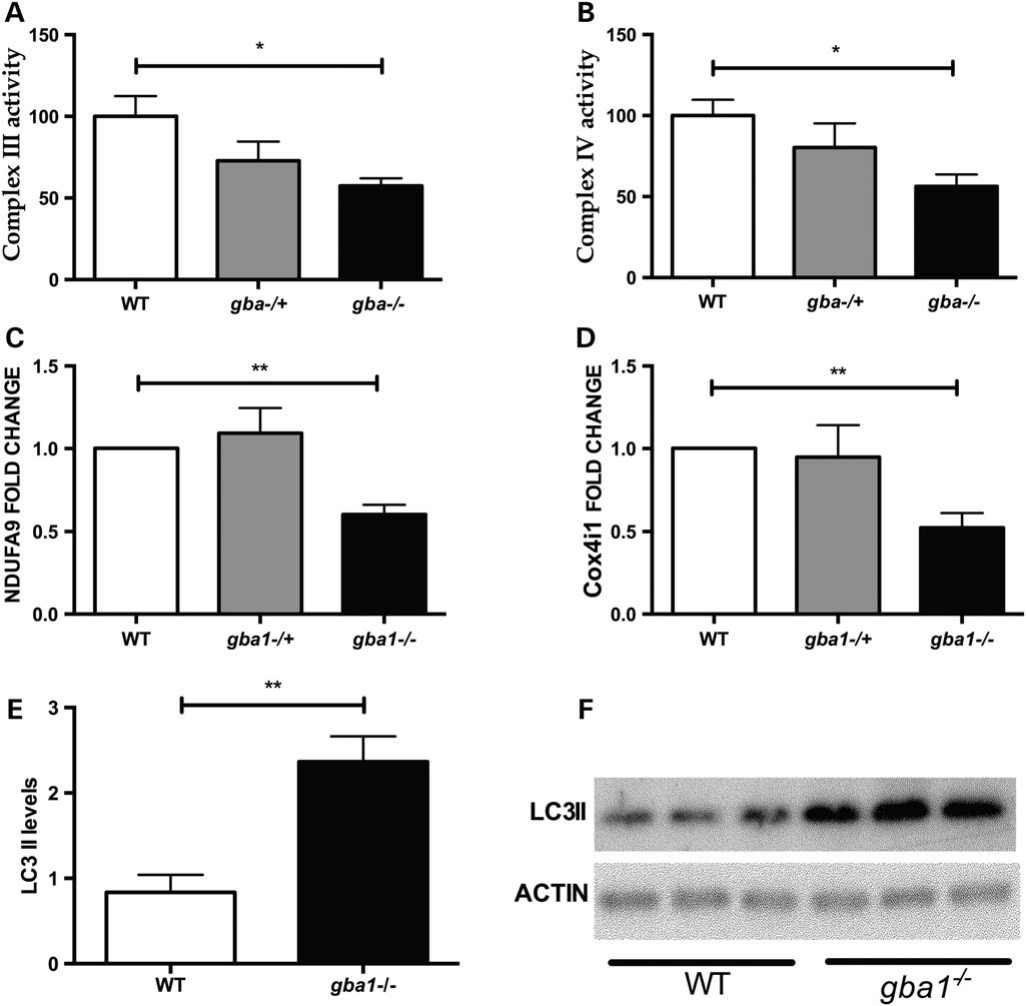
Mitochondrial dysfunction and autophagy in *gba1^−/−^* zebrafish. There was a 50% decrease (*P* < 0.05) in complex III (**A**) and complex IV (**B**) activity in *gba1^−/−^* brains at 12 wpf. There was also a decrease in the protein levels of the complex I subunit NDUFA9 (**C**, *P* < 0.01) and the complex IV subunit Cox4i1 (**D**, *P* < 0.01). LC3-II was increased 2-fold in *gba1^−/−^* brains at 12 wpf compared with wild-type (WT) (**E**, *P* < 0.01). (**F**) A representative western blot of LC3-II protein levels in wild-type and *gba1*^−/−^ zebrafish brains. *P < 0.05; **P < 0.01.

Mitochondrial dysfunction has been demonstrated in other models of *gba1* deficiency (23). We analyzed the activity of the mitochondrial respiratory chain in 12 wpf brain tissue across the *gba1* genotypes. Complex III and IV activity was lower by ∼50% in *gba1*^−/−^ compared with wild-type (*P* < 0.05). Both complex III and IV activity in *gba1*^+/−^ fish had intermediate values between those seen in *gba1*^−/−^ and wild-type, but did not differ significantly from either (Fig. 8A and B). We hypothesized that the observed specific abnormalities in mitochondrial function seen in *gba1*^−/−^ fish may be due to impaired mitochondrial biogenesis or mitochondrial protein turnover, possibly linked to impaired mitophagy. However, the outer mitochondrial membrane protein TOMM20 and TIMM9 (located in the inter membrane space) levels were similar across the three genotypes (data not shown). In contrast, NDUFA9 (encoding a complex I subunit) and Cox4i1 (encoding a complex IV subunit) were reduced in *gba1*^−/−^ brains compared with controls (Fig. 8C and D, *P* < 0.01). The reduction of Cox4i1 may at least in part underlie the observed lowering of complex IV activity. ATP5A (encoding a complex V subunit) was also somewhat lower in *gba1*^−/−^ fish but this difference was not significant (*P* > 0.05; data not shown).

GCase deficiency results in lysosomal dysfunction due to the accumulation of its substrate, glucocerebroside and in mice lacking *Gba1*, decreased autophagosome formation and accumulation of autophagy substrates in the brain as well as decreased mitophagy has been observed (23). Therefore, we investigated whether autophagy was disrupted in the brains of 12 wpf *gba1* mutants compared with wild-type siblings. LC3-II is specifically targeted to autophagosomal membranes and strongly correlates with the number of autophagosomes (24). Brains from *gba1*^−/−^ fish had more than 2-fold increase in LC3-II levels compared with wild-type siblings (Fig. 8E and F; *P* < 0.01). Whereas this difference in LC3-II levels clearly demonstrates that autophagosome number is altered in *gba1*^−/−^ brains compared with those of wild-types, it is unclear whether autophagosome formation is increased or whether autophagosome degradation is defective, because both of these scenarios would lead to an increase in LC3-II levels.

## Discussion

Modern gene editing techniques such as the TALEN strategy have transformed zebrafish research (25). We have used the TALEN approach to generate a *gba1* mutant zebrafish line which faithfully resembles key pathological and biochemical features of human GCase deficiency. We provide data on *gba1^−/−^* mRNA stability, reduced GCase enzymatic activity and other biochemical readouts including extensive mass spectrometry-based analysis of sphingolipids which all support the presence of a marked biological effect caused by the TALEN-induced 23 bp deletion in *gba1* on GCase function.

This zebrafish model of GCase deficiency is the first vertebrate model to faithfully replicate key GD pathology in both visceral and neural tissue simultaneously. Conventional KO and conditional KO mice model either neuropathic or non-neuropathic Gaucher disease but not both (10). Our extensive glycolipid mass spectrometry analysis suggests that it is mostly lower MW species which accumulate, with high MW species either unchanged or decreased compared with wild-type. The predominant increase of C18 metabolites is in keeping with similar studies in other model systems (16,26,27). Our observation of a marked increase in the accumulation of distinct glycosphingolipids prior to the onset of marked inflammation and neuronal cell loss in GCase deficient zebrafish larvae is in keeping with similar observations in a mouse model of neuronopathic GD (14). miR-155 is a master regulator of pathways involved in the regulation of immune mechanisms (21) that is expressed in both the innate and the adaptive immune system and predominantly acts via moderate mRNA degradation. Of note, miR-155 upregulation has already been implicated in the pathogenesis of different neurodegenerative disorders. Early miR-155 upregulation contributes to neuroinflammation in an Alzheimer's disease transgenic mouse model as well as in Aβ-activated microglial and astrocyte cultures (28). Expression levels of miR-155 are increased in the spinal cord of both familial and sporadic amyotrophic lateral sclerosis and genetic ablation of miR-155 markedly increased survival in SOD1 mice with restoration of abnormal microglia (29). However, miR-155 had not been implicated in the pathogenesis of GD or PD before now. Our study clearly suggests that activation of immune mechanisms precedes neuronal cell loss rather than being a consequence of it. Future work needs to determine whether miR-155 may also be a promising ‘druggable’ target for neuroprotective therapy in both GD and PD. Of note, an association of *GBA* mutation status with an increase in the plasma levels of different inflammatory mediators such as interleukin 8 has been reported in PD patients (30).

Both loss of GCase function and toxic gain of function have been proposed to explain the increased risk of PD for *GBA1^+/−^* carriers (6,31). There is also strong evidence for an interplay between GCase activity and alpha-synuclein levels (9,16,32). The marked loss of dopaminergic neurons in *gba1^−/−^* zebrafish in the absence of alpha-synuclein indicates that alpha-synuclein-independent mechanisms can contribute to the neurodegeneration resulting from GCase deficiency. The extensive accumulation of ubiquitin-positive intra-neuronal inclusions in the brains of juvenile *gba1^−/−^* zebrafish further suggests that proteins other than alpha-synuclein accumulate in this model. Obvious candidates are the β- and γ1-synucleins expressed in the zebrafish CNS (22). However, western blot analysis showed that these are both markedly reduced in juvenile *gba1*^−/−^ brains, possibly as a consequence of extensive synaptic loss accompanying neurodegeneration in this model. Indeed, neuronal ubiquitinopathy preceding an increase in alpha-synuclein levels has been described in a *GBA1* knock-in mouse model (33) and it is likely that *gba1^−/−^* zebrafish represent an example of non-synuclein proteinopathy and synuclein-independent neurodegeneration occurring in the absence of GCase activity.

Mitochondrial dysfunction with impaired quality control has been reported in a mouse model of GD and iPSC-derived *GBA1^+/−^* neurons (16,23). However, we observed reduced complex III and IV activity rather than reduced complex I activity as observed in *GBA1^−/−^* mice (23). We hypothesize that this may at least in part be due to alpha-synuclein-mediated mitochondrial toxicity in *GBA1^−/−^* mice which typically affects complex I activity (34). The reduced complex IV activity in *gba1^−/−^* juvenile zebrafish brains may be due to a direct effect of the markedly elevated glucosylsphingosine, a potent inhibitor of the mitochondrial cytochrome *c* oxidase on the environment of this membrane-bound enzyme (35). Interestingly, magnetic resonance spectroscopic imaging data in human patients also provide circumstantial evidence of altered membrane phospholipid metabolism in *GBA1*-associated PD (36). Alternatively, the reduced complex IV activity may at least partially be due to the observed reduction in the Cox4i1 protein level the *gba1^−/−^* brains (Fig. 8D). Autophagy plays an essential role in the clearance of aggregate-prone proteins and damaged mitochondria, and dysfunctional autophagy has been implicated in the pathogenesis of PD (37). In mouse models of GCase deficiency, autophagosome formation is decreased and ubiquitylated proteins monomeric and oligomeric forms of alpha-synuclein and ubiquitylated proteins accumulate in the brain (23). In juvenile *gba1^−/−^* zebrafish brains, we observed a significant increase in LC3-II levels which may result from either an increase in autophagosome formation or a defect in degradation.

Some of our findings are remarkably similar to observations in a *GBA1* nonsense medaka (*Oryzias latipes*) model of Gaucher disease (27). Future studies need to reveal whether the observed early microglial activation and subsequent neuronal cell loss is linked to the recently reported Wnt signaling abnormalities in GCase1 deficient *D. rerio* zebrafish with reduced CGase activity caused by transient antisense knockdown of *gba1* early in development (38).

## Conclusion

Zebrafish are an excellent vertebrate model to study human brain diseases and increasingly used for high-throughput drug screens (39,40). The large sphingolipid accumulation and microglial dysfunction during larval stages shows the potential to use the *gba1* mutant zebrafish as a tool for phenotypic drug discovery to identify new disease modifying therapies for neuronopathic GD and to aid in the identification of novel PD toxins that may act synergistically in conjunction with *gba1^+/−^.* There is growing evidence of lysosomal impairment in PD in general and decreased activity of GCase in particular, even in the absence of *GBA1* mutations (32,41–44). Augmenting CNS GCase activity has been proposed as a promising therapeutic strategy for PD and other GD-related synucleinopathies (45). A further promising aim for zebrafish *in vivo* high-throughput screens could therefore be to identify compounds which would upregulate neuronal GCase activity.

## Materials and Methods

### Zebrafish husbandry

All larval and adult zebrafish were housed at the University of Sheffield; experimental procedures being in accordance with UK Home Office Animals (Scientific Procedures) Act 1986 (Project license PPL 70/8437, held by Dr Oliver Bandmann). Adult zebrafish were housed at a density of 40 per tank, whereas on a cycle of 14 h of light, 10 h of dark. Adults and embryos were kept at constant temperature of 28°C.

### *gba1* stable mutant line

A stable loss of function allele was generated with the TALEN genome editing system targeting an *mwo*I restriction enzyme site located within exon seven of *gba1*. A pair of TALENs binding 5′TCTGTACCCTGATTACTT (right TALEN) and 5′ATGCGCTGGGTGGAGTCCA (left TALEN) were chosen by the TALEN targeter (https://boglab.plp.iastate.edu/node/add/talen). TALEN mRNA was generated and injected into one cell stage Zebrafish embryos. F0 mosaic founders were identified and outcrossed to wild-type TL adults. A (heterozygous) allele was identified in the F1 generation containing a 23 bp deletion (*gba1^+/−^*) and outcrossed again to TL until the F3 generation was reached. Zebrafish homozygous for this 23 bp deletion (*gba1^−/−^*) used for all experiments were generated from an incross of F3 *gba1^+/−^*. All zebrafish were genotyped using primers F-5′AAAGCAGCACGATATGTCCA and R-5′ATGTCATGGGCGTAGTCCTC. DNA was amplified and analyzed on a 2% agarose gel.

### Gene expression analysis

RNA was extracted from 20 zebrafish embryos/zebrafish caudal hypothalamus by 40% (*P* < 0.01; Fig. 7B) and the posterior tuberculum by ∼30% (*P* < 0.01; Fig. 7C). brains (two per replicate) at specific time points using TRIzol^®^ (Life Technologies™). A Verso cDNA synthesis kit (Thermo Scientific) was used to generate cDNA. Quantitative real-time PCR (qPCR)-based quantification of *gba1* expression was undertaken using primers F-5′GGCACAGGCTCTATCTGCTC and R-5′TCTAGAAACCTGATATAGT. SYBR (Life Technologies™) green was used for all qPCR experiments, with *ef1a* as a reference gene (*ef1a* primers: F-5′TGGTACTTCTCAGGCTGACT and R-5′TGACTCCAACGATCAGCTGT). For microRNA expression analysis, RNA was harvested from embryos and brain tissue as previously described. RNA concentration was accurately quantified using the QuantiFluor™ RNA system (Promega) and the Qubit^®^ fluorometer (Life Technologies). 100 ng of total RNA was reverse-transcribed and subsequently qPCR was performed using Taqman miRNA assays (Applied Biosystems). A Taqman probe (sequence: 5′UUAAUGCUAAUCGUGAUAGGGG) was used to quantify miR-155 levels.

### Lysosomal enzyme analysis, assessment of mitochondrial respiratory chain function and mass spectrometry

All lysosomal enzyme assays were performed on homogenates of whole zebrafish brain at 12 wpf with a protein concentration of 1 mg/ml and at 28°C unless otherwise stated. All assays were stopped with 1 M glycine NaOH buffer pH 10.4 and used 1 nm 4-methylumbelliferone (Sigma) as a standard to calculate the final result. Chitotriosidase activity was measured using 4-methylumbelliferyl-β-d-*N*,*N*′,*N*″-triacetyl-chitotriose (Sigma) in McIlvaine citrate–phosphate buffer pH 5.2. β-Hexosaminidase activity was measured using 4-methylumbelliferyl-2-acetamido-2-deoxy-β-d-gluco-pyranoside (Sigma) in McIlvaine citrate–phosphate buffer pH 4.5. Beta-galactosidase activity was measured using 1 mm 4-methylumbelliferyl-d-galactopyranoside dissolved in McIlvaine citrate–phosphate buffer pH 4.1. GCase activity was measured with 5 mm 4-methylumbelliferyl-β-d-glucopyransoside (Sigma) in McIlvaine citrate–phosphate buffer pH 5.4 in the presence and absence of conduritol B epoxide at 37°C.

Mitochondrial complex activities I–IV were assessed in whole brain homogenates at 12 wpf as previously described (46).

Mass spectrometry for the detection of sphingolipid metabolites was undertaken at 5 dpf and 12 wpf as previously described (47). Larvae were genotyped as previously described (48). Genotype larvae were then frozen in liquid nitrogen in groups of 20 per genotype (wild-type, *gba1^+/−^* and *gba1^−/−^*) and stored at −80°C prior to mass spectrometric analysis. Mass spectrometry was then also undertaken in brains of juvenile zebrafish (12 wild-type, 10 *gba1^+/−^* and 10 *gba1^−/−^*) at 12 wpf.

### Assessment of dopaminergic nervous system

Dopaminergic neurons were first counted at 5 dpf using whole mount *in situ hybridization* (WISH) staining with a probe for *tyrosine hydroxylase* (TH) (*n* = 10 embryos per genotype and biological replicate). Dopaminergic neurons were counted by eye using an axioplan compound microscope (Zeiss) at 20× magnification as previously described (46). The counter was blinded to the genotype and condition. The dopaminergic neuron count was assessed by counting the distinct neuronal subgroups one, two, four and five in the diencephalon, defined according to the Rink and Wullimann classification (49,50). The mean neuron count of each control group was normalized to 100% and all other group counts expressed as a percentage of the control group. Juvenile zebrafish were culled and brains fixed in paraformaldehyde (PFA) to enable dopaminergic neuronal cell count at 12 wpf in wild-type, *gba1^+/−^* and *gba1^−/−^* zebrafish. Dopaminergic neurons were stained using a TH1 antibody (Mouse monoclonal anti-TH, DiaSorin Inc.) and then counted in the posterior tuberculum and caudal hypothalamus as previously described (51).

### Movement analysis

Locomotion was quantified using Viewpoint analysis software version 3, 22, 3, 9 (Viewpoint). Fish were filmed individually from the side, for 10 min following 10 min acclimation time. Low-speed movements were defined as <5 cm/s. Medium-speed movements were defined as 5< *X* <7 cm/s. High-speed movements were defined as movements >7 cm/s.

### Microglial activation

*gba1^+/−^* were crossed to *Tg*(*mpeg1:GFP-CAAX*) (*mpeg1*,sh425), similar to a previously published protocol (52). Details on the transgenesis methods are available from the authors. All subsequent embryo work was generated from an incross of *gba1^+/−^* and *mpeg1* and imaged at 4 dpf. High-resolution imaging was performed using an inverted UltraViewVoX spinning-disk confocal microscope (PerkinElmer Life and Analytical Sciences). Imaging was performed to a depth of ∼150 µm from the dorsal surface of the brain using 2 µm z-sections. Volumetric and shape factor analyses were performed using Volocity 6.3 (PerkinElmer Life and Analytical Sciences) software, using intensity of fluorescence to identify individual cells. Measurements of vacuole diameter were performed manually using the line tool. Data for the assessment of microglial shape and vacuole count were pooled form three independent experiments including a total of 177 wild-type, 94 *gba1^+/−^* and 82 *gba1^−/−^* microglial cells from 15 wild-type, 9 *gba1^+/−^* and 7 *gba1^−/−^* larvae. All measurements were performed blind to the *gba1* genotype. Following microscopy and image analysis, embryos were genotyped for the *gba1* mutation (as described earlier).

### Histology

For H&E and PAS staining, zebrafish were fixed in Bouins fixative for 2 weeks and embedded in paraffin. Ubiquitylation was assessed in zebrafish fixed in 10% buffered formalin solution for 1–2 weeks with subsequent decalcification for 7 days in ethylenediaminetetraacetic acid. Coronal or sagittal sections were made of ∼4 μm thickness. Each zebrafish was sectioned completely and every 10th and 11th slide was used for subsequently staining with either H&E or PAS. Ubiquitin immunohistochemistry (IHC) was performed with antigen retrieval by pressure cooker at pH 6, using a polyclonal anti-ubiquitin antibody (Dako Z 0458) at a 1:1000 dilution, standard ABC methods and diaminobenzidine (DAB) as chromogen. Prepared microscope slides were viewed by a board-certified pathologist (A.M.), using conventional bright-field microscopy.

Sample preparation for IHC and confocal microscopy to investigate microglial activation in juvenile zebrafish brains was carried out as reported previously (53). Zebrafish were perfused and brains post-fixed in 4% PFA, followed by cryoprotection in PBS-sucrose. Fourteen micrometer thick cryosections were mounted on glass slides, treated with PBS-T (0.3% Triton-X) for 1 h, blocked with 10% goat serum in PBS for 2 h and then incubated overnight at 4°C with primary antibodies diluted 1:20 (7.4.C4, purified from hybridoma clone; #92092321, HPA Culture Collections, UK), 1:500 (P0) in PBS with 1% goat serum (54). Primary antibodies were detected using Alexa-488 (anti-mouse), and Alexa-555 (anti-rabbit) conjugated secondary antibodies (Life Technologies, Grand Island, NY) diluted 1:1000 in carrier buffer and sections counter labeled with 4′, 6-diamidino-2-phenylindole (DAPI). Images were acquired using an Olympus Fluoview confocal microscope and multi-field collages made with Adobe Photoshop.

### Western blotting

Mitochondrial primary antibodies: TOMM20 (Santa Cruz), TIMM9 (Abcam), NDUFA9 (Abcam), COX4i1 (Abcam), ATP5A (Abcam), β-ACTIN (Sigma-Aldrich). Horseradish peroxidase (HRP)-linked secondary antibodies were used (Sigma-Aldrich). LC3 primary antibodies: Rabbit anti-LC3 (Novus Biologicals; NB100–2220) used at 1:1000 dilution; mouse anti-actin (Sigma A5316) used at 1:500 dilution. Secondary antibodies: Polyclonal goat anti-rabbit immunoglobulins/HRP (Dako P0448) used at 1:5000; polyclonal goat anti-mouse immunoglobulins/HRP (P0447) used at 1:5000. Beta- and Gamma-synuclein primary antibodies: mAb α/β-Synuclein (Syn205, Cell Signaling; 1:1000) or pAb γ1-Synuclein (1:1000). γ1-Synuclein polyclonal antibody was raised to the peptide DFSHGGMEGGEGGEGY by immunization of rabbits (New England Peptide) and affinity purified as previously described (54). IRDye-800 and IRDye-680 (LI-COR, Lincoln, NE) conjugated secondary antibodies (1:10 000) enabled the blot to be imaged using an Odyssey Infrared Imager (catalog no. 9120; LI-COR) with a wide linear range.

### Statistical tests and analysis

Graphpad prism V.5 software (Graphpad) was used for statistical analysis and all errors bars shown denote the mean ± SE of the mean. All experiments were performed in biological triplicate unless otherwise. All data were analyzed with either *T* test, one-way ANOVA or two-way ANOVA.

## Supplementary Material

Supplementary Material is available at *HMG* online.

## Funding

This work was supported by BBSRC/Lilly (PhD CASE studentship for M.K.); Parkinson's UK (G-1404 for O.B.); The Academy of Finland and Sigrid Juselius Foundation (for P.P.); FishMed/EU Seventh Framework Programme (no. 316125 for A.S.); Wellcome Trust/MRC Joint call in Neurodegeneration award to the UK Parkinson’s Disease Consortium (WT089698 for M.G. and A.H.V.S.); National Institute for Environmental Health Sciences (ES022644 for E.A.B.); Australian NHMRC C.J. Martin fellowship to F.E. (GNT1054664), MRC Programme Grant (SAR; reference number MR/M004864/1), MRC Center grant (G0700091), University of Sheffield Vice-Chancellor's Fellowship (PME). Microscopy studies were supported by a Wellcome Trust grant to the MBB/BMS Light Microscopy Facility (GR077544AIA). Funding to pay the Open Access publication charges for this article was provided by Parkinson’s UK.

## Supplementary Material

Supplementary Data
