# Supplementary material for: Glucocerebrosidase 1 deficient Danio rerio mirror key pathological aspects of human Gaucher disease and provide evidence of early microglial activation preceding alpha-synuclein-independent neuronal cell death
Source: Hum Mol Genet. 2015 Sep 16;24(23):6640–52. doi: 10.1093/hmg/ddv369 (PMC4634372; doi:10.1093/hmg/ddv369)
Supplement: Supplementary Data [file supp_24_23_6640__index.html]

Glucocerebrosidase 1 deficient Danio rerio mirror key pathological aspects of human Gaucher disease and provide evidence of early microglial activation preceding alpha-synuclein-independent neuronal cell death — Glucocerebrosidase 1 deficient Danio rerio mirror key pathological aspects of human Gaucher disease and provide evidence of early microglial activation preceding alpha-synuclein-independent neuronal cell death — Glucocerebrosidase 1 deficient Danio rerio mirror key pathological aspects of human Gaucher disease and provide evidence of early microglial activation preceding alpha-synuclein-independent neuronal cell death — Supplementary Data 

# Glucocerebrosidase 1 deficient *Danio rerio* mirror key pathological aspects of human Gaucher disease and provide evidence of early microglial activation preceding alpha-synuclein-independent neuronal cell death

## Supplementary Data

Supplementary Data

- Supplementary Data - Docx file
- Supplementary Table 1 - xlsx file
- Supplementary Figure 1 - tif file
- Supplementary Video 1 - MOV file
