# Supplementary material for: Glucocerebrosidase 1 deficient Danio rerio mirror key pathological aspects of human Gaucher disease and provide evidence of early microglial activation preceding alpha-synuclein-independent neuronal cell death
Source: Hum Mol Genet. 2015 Sep 16;24(23):6640–52. doi: 10.1093/hmg/ddv369 (PMC4634372; doi:10.1093/hmg/ddv369)
Supplement: Supplementary Data [file supp_ddv369_ddv369supp.docx]

**Supplementary figure 1**. Location of TALEN mutation within *gba1*. The black boxes depict the separate exons. The entire (wild type) DNA sequence of exon 7 is given (black letters). The red letters depict the base pairs deleted in the *gba1*^-/-^ mutant zebrafish (*gba1^c.1276_1298del^*). The location of the induced premature stop codon resulting from the 23 bp deletion is highlighted in yellow.

**Supplementary table 1.** Mass spectrometric analysis of sphingolipid metabolites in both 5dpf larvae and brain tissue at 12wpf in *gba1^-/-^* compared to WT. Changes in *gba1*^-/-^ samples are expressed as a percentage change to WT. Increased levels are depicted in red, decreases in green, no changes in yellow. No significant changes were detected between *gba1^+/-^* and wt for either 5 dpf larvae or brain tissue at 12 wpf (data not shown).

**Supplementary video 1.** Example of “barrel” rolling phenotype exhibited by *gba1^-/-^* at 12 wpf.
