# Supplementary figures and images for: Glucocerebrosidase 1 deficient Danio rerio mirror key pathological aspects of human Gaucher disease and provide evidence of early microglial activation preceding alpha-synuclein-independent neuronal cell death
Source: Hum Mol Genet. 2015 Sep 16;24(23):6640–52. doi: 10.1093/hmg/ddv369 (PMC4634372; doi:10.1093/hmg/ddv369)

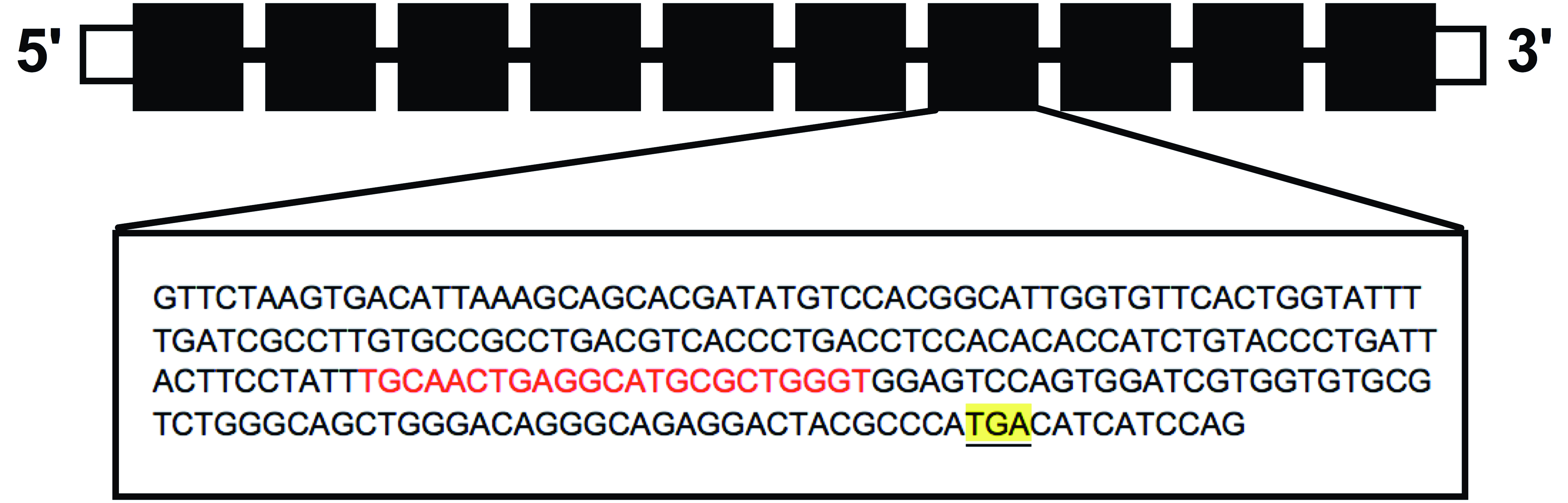

Supplement: Supplementary Data [file supp_ddv369_ddv369supp_fig1.tif]
